# A single amino acid residue substitution in *BraA04g017190.3C*, a histone methyltransferase, results in premature bolting in Chinese cabbage (*Brassica rapa* L. ssp. *Pekinensis*)

**DOI:** 10.1186/s12870-021-03153-9

**Published:** 2021-08-13

**Authors:** Chong Tan, Jie Ren, Lin Wang, Xueling Ye, Wei Fu, Jiamei Zhang, Meng Qi, Hui Feng, Zhiyong Liu

**Affiliations:** Liaoning Key Laboratory of Genetics and Breeding for Cruciferous Vegetable Crops, Department of Horticulture, Shenyang Agricultural University, 110866 Shenyang, People’s Republic of China

**Keywords:** *Brassica rapa*, Flowering, MutMap, CURLY LEAF, EMS mutagenesis

## Abstract

**Background:**

Flowering is an important inflection point in the transformation from vegetative to reproductive growth, and premature bolting severely decreases crop yield and quality.

**Results:**

In this study, a stable early-bolting mutant, *ebm3*, was identified in an ethyl methanesulfonate (EMS)-mutagenized population of a Chinese cabbage doubled haploid (DH) line ‘FT’. Compared with ‘FT’, *ebm3* showed early bolting under natural cultivation in autumn, and curled leaves. Genetic analysis showed that the early-bolting phenotype was controlled by a single recessive nuclear gene. Modified MutMap sequencing, genotyping analyses and allelism test provide strong evidence that *BrEBM3* (*BraA04g017190.3 C*), encoding the histone methyltransferase CURLY LEAF (CLF), was the strongly candidate gene of the *emb3*. A C to T base substitution in the 14th exon of *BrEBM3* resulted in an amino acid change (S to F) and the early-bolting phenotype of *emb3*. The mutation occurred in the SET domain (Suppressor of protein-effect variegation 3–9, Enhancer-of-zeste, Trithorax), which catalyzes site- and state-specific lysine methylation in histones. Tissue-specific expression analysis showed that *BrEBM3* was highly expressed in the flower and bud. Promoter activity assay confirmed that *BrEBM3* promoter was active in inflorescences. Subcellular localization analysis revealed that BrEBM3 localized in the nucleus. Transcriptomic studies supported that *BrEBM3* mutation might repress H3K27me3 deposition and activate expression of the *AGAMOUS* (*AG*) and *AGAMOUS*-*like* (*AGL*) loci, resulting in early flowering.

**Conclusions:**

Our study revealed that an EMS-induced early-bolting mutant *ebm3* in Chinese cabbage was caused by a nonsynonymous mutation in *BraA04g017190.3 C*, encoding the histone methyltransferase CLF. These results improve our knowledge of the genetic and genomic resources of bolting and flowering, and may be beneficial to the genetic improvement of Chinese cabbage.

**Supplementary Information:**

The online version contains supplementary material available at 10.1186/s12870-021-03153-9.

## Background

Flowering is a crucial developmental process that marks the transition from vegetative to reproductive growth, and is essential for propagation. The timing of floral induction is determined by the interaction of environmental and endogenous cues, ensuring that flowering occurs under the conditions the most likely to maximize offspring quantity and quality [1, 2]. One goal of plant breeding is to improve plant adaptability to climate changes and new environment by controlling flowering time, to ultimately increase crop yield and quality. An enhanced understanding of the flowering regulatory network is of great value for crop genetic selection and improvement.

The genetic control of flowering time, including its interwoven network, has been extensively studied in the long-day (LD) model *Arabidopsis thaliana* and short-day (SD) model plant rice (*Oryza sativa*) [3]. In *A.thaliana*, six major genetic pathways controlling flowering time, i.e., photperiod, vernalization, autonomous, gibberellin, ambient temperature, and age, have been described [4]. Flowering is one of the most complex regulated pathways, the signaling cross-talk between the pathways induced flowering is ubiquitous. More specifically, such as cross-talk between vernalization and photperiod pathways ensures that plants adapt more effectively during unpredictable environmental condidtions [5]. In the floral induction phase, these pathways converge on floral integrator genes to control flowering time, thereby activating the downstream meristem identity genes. *FLOWERING T* (*FT*) and *SUPPRESSOR OF OVEREXPRESSION OF CONSTANS 1/ AGAMOUS-LIKE 20* (*SOC1*/*AGL20*) are the two key floral integrator genes [6]. FT, which belongs to the phosphatidylethanolamine-binding protein (PEBP) family, has two homologs, namely TWIN SISTER OF FT (TSF) and TERMINAL FLOWER 1 (TFL1). And TSF and TFL1 act redundantly and antagonistically to FT, respectively. FT is produced in the leaves and is transported via the phloem to the shoot apical meristem (SAM), where it interacts with FLOWERING LOCUS D (FD) to induce *SOC1* and the floral meristem identify genes *APETALA 1* (*AP1*) and *CAULIFLOWER* (*CAL*) [7].

The photoperiod and vernalization pathways control flowering in response to seasonal changes in day length and temperature [8]. In the photoperiod pathway, *CONSTANS* (*CO*) is the main positive regulator of *FT*/*TSF*. *TEMPRANILLO* (*TEM*) genes are reported to have a pivotal role in the direct repression of *FT* and counteract the activator *CO* [9]. *CYCLING DOF FACTORs* (*CDFs*) are transcriptional repressors of *CO.* FLAVIN-BINDING KELCH REPEAT F-BOX 1 (FKF1) and GIGANTEA (GI) form a stable complex that releases repression of *CO* by inducing degradation of *CDF1* [10]. At the posttranscriptional level, CONSTITUTIVE PHOTOMORPHOGENIC 1 (COP1) and SUPPRESSOR OF PHYTOCHROME A (SPA1) form a ubiquitin ligase complex that facilitates CO degradation in the dark [8]. LATE ELONGATED HYPOCOTYL (LHY) and CIRCADIAN CLOCK-ASSOCIATED1 (CCA1), are two MYB protein that play important roles in photoperiod pathway by controlling the rhythmic expression of flowering-time genes [11]. In the vernalization pathway, *FLOWERING LOCUS C* (*FLC*), which encodes a MADS-box transcription factor, acts as a central floral repressor by directly repressing the transcription of the floral promoting genes *FT, SOC1/AGL20*, and *SQUAMOSA PROMOTER BINDING PROTEIN-LIKE 15* (*SPL15*) [12]. *FRIGIDA* (*FRI*), which encodes a coiled-coil protein, positively regulates *FLC* by affecting its chromatin structure [13]. *VERNALIZATION INSENSITIVE 3* (*VIN3*), which encodes a PHD-finger protein, is necessary for epigenetic silencing of *FLC* [14]. Two long noncoding RNAs (lncRNAs), cold-induced long antisense RNA (COOLAIR) and cold-assisted intronic noncoding RNA (COLDAIR), are responsible for transcriptional shutdown of *FLC* [15, 16]. MADS AFFECTING FLOWERING (MAF), a member of FLC-like protein, can form protein complexs with FLC [17]. In addition, *AGL19* has been found to promote flowering with interacting with FLC in vernalization pathway[18].

The effects of the autonomous, age, and gibberellin pathways are more independent of environmental stimuli. In the autonomous pathway, *FCA*, *FY*, *FPA*, *FVE*, *FLOWERING LOCUS D* (*FLD*), *FLOWERING LOCUS KH DOMAIN* (*FLK*), and *LUMINIDEPENDENS* (*LD*) participate in repressing *FLC* to accelerate flowering [8]. In the age pathway, miR156-targeted *SPL* transcription factors and miR172-targeted *APETALA 2* (*AP2*) and *AP2*-*like* genes are the two main modules [19]. *AP2* and *AP2*-*like* genes inhibit the onset of flowering by repressing expression of (*FRUITFULL*) *FUL*/*AGL8*, *SCO1*/*AGL20*, and *AG* [20]. In *A. thaliana*, DELLAs are master negative regulators of gibberellin (GA) signal transduction. Upon binding to GA, GIBBERELLIN INSENSITIVE DWARF1 (GID1) undergoes a conformational change, which creates a surface for the binding of DELLAs to form a GA-GID1-DELLA complex. DELLAs are then recruited to E3 ubiquitin ligase SCF^SLY1/GID2^ for polyubiquitination, leading to the degradation of DELLAs by the 26 S proteasome [21]. Furthermore, GA 20-oxidases (GA20ox1-5), GA 3-oxidases (GA3ox1-3), and GA 2-oxidases (GA2ox1-5) are involved in the GA pathway [8].

The ambient temperature pathway controls flowering in response to the daily growth temperature. *SHORT VEGETATIVE PHASE* (*SVP*) plays a key role in responding to changes in ambient temperature [22].

*Brassica rapa* closely related to *A. thaliana*, both belong to Brassicaceae family [8]. *B. rapa* shares *A. thaliana’s three* paleo-polyploidy events (γ, β, and α), and experiences an additional whole genome triplication (WGT) event since the divergence from the last common ancestor with *A. thaliana* [23]. Thus, the genetic pathways controlling flowering time in *B. rapa* are more complex than those in *A. thaliana* because there are multiple copies of paralogs. Two paralogs of FT (*BrFT1* and *BrFT2*) are found in *B. rapa*, and a transposon insertion in *BrFT2* induces late flowering [24]. Three paralogs of *SOC1* (*Br004928*, *Br000393*, and *Br009324*) are expressed, and at least two of them have been predicted to play a role in flowering in a natural population [25]. Overexpression of *BrSOC1*/*BrAGL20* in *B. napus* results in early flowering [26]. *B. rapa* comprises four *FLC* genes, namely, *BrFLC1*, *BrFLC2*, *BrFLC3*, and *BrFLC5* [27]. *BrFLC1*, *BrFLC2*, and *BrFLC3*, syntenic orthologs of *AtFLC*, have been found to negatively regulate flowering [28–33]. A recent study has shown that *BrFLC5* is a weak regulator of flowering time [34]. Two *FRI* paralogs in *B. rapa*, *BrFRIa* and *BrFRIb*, are activators of *BrFLC* [29]. In vernalized *B. rapa*, DNA demethylation of two subunits of casein kinase II (CK2), *BrCKA2* and *BrCKB4*, shortens the period of *BrCCA1* [35]. In *B. rapa*, a pakchoi *MAF* gene, *BcMAF1*, delays flowering by directly activating *BcMAF2* and repressing *BcAP3* [36]. *BcMAF2* can directly activate *BcTEM1* and repress flowering [37]. Two *B. rapa GI* alleles are responsible for rescuing the late-flowering phenotype of an *Arabidopsis gi-201* mutant [38].

Chinese cabbage is the most leafy *B. rapa* crop in East-Asian countries, composed of a large number of tightly wrapped heading leaves [39]. Flowering time is an important agronomic trait for Chinese cabbage, and premature bolting can severely reduce crop yield and quality. In the present study, we characterized an early-bolting Chinese cabbage mutant identified from an EMS-mutagenized population. By performing MutMap sequence, kompetitive allele-specific polymerase chain reaction (KASP) analyses, and allelism test, a nonsynonymous base substitution in *BrEBM3* was identified to cause the mutant phenotype. The expression pattern of the candidate gene *BrEBM3* was comprehensively analyzed by evaluating spatiotemporal expression, promoter activity, and subcellular localization. Transcriptome profiling was conducted to identify potential *BrEBM3*-regulated genes responsible for flowering time in Chinese cabbage. We expected our findings to be of great significance for further study of the molecular mechanism of bolting and flowering in Chinese cabbage.

## Results

### Morphological characteristics and genetic analysis of the mutant *ebm3*

**Fig. 1:**
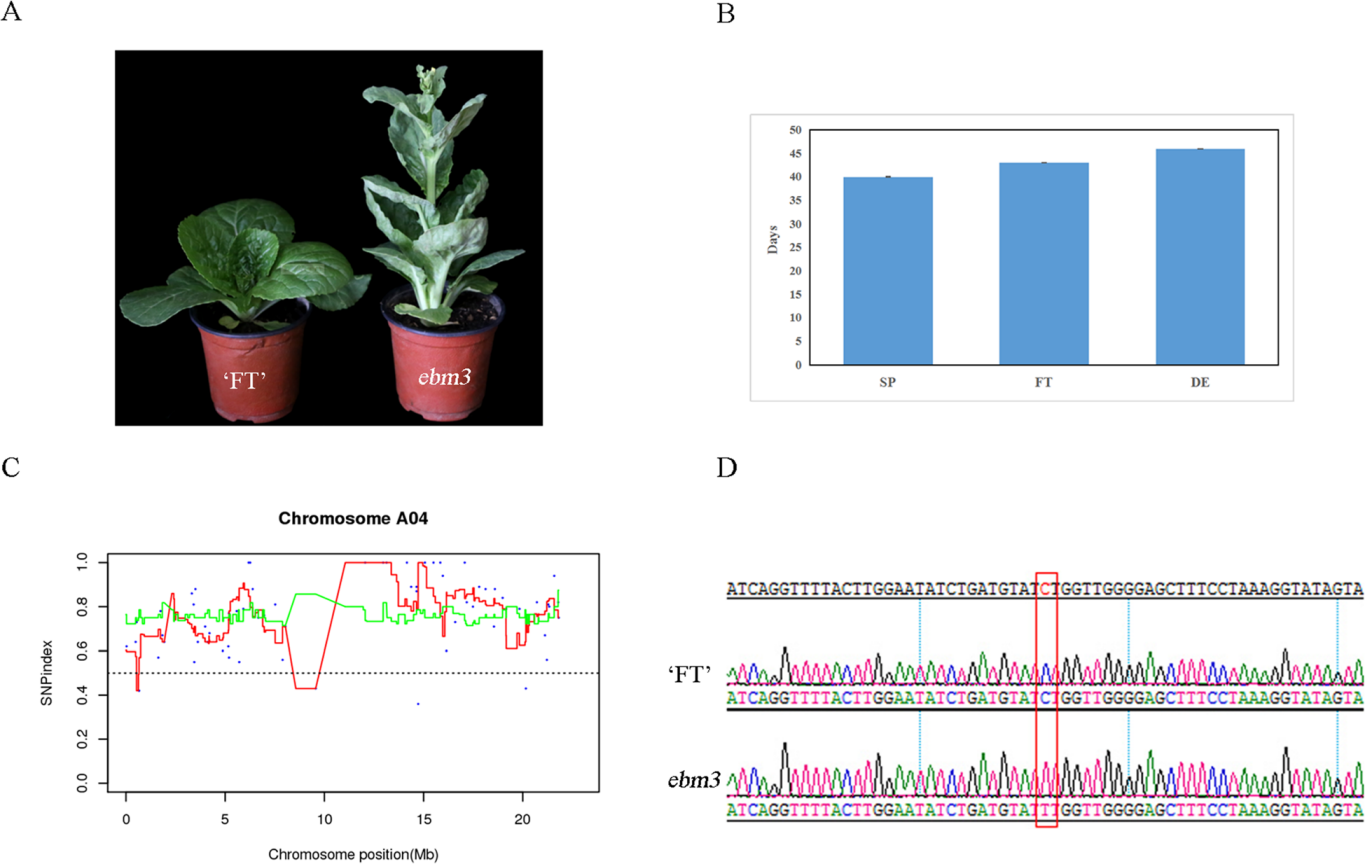
Identification of the mutant *ebm3* and candidate SNPs. **a** Phenotypic characterization of the wild-type line ‘FT’ (left) and the mutant *ebm3* (right). The plants were photographed under DS-Fi3 microscope camera (Nikon Corporation, Japan). **b** Phenotypic values for mutant *ebm3* under normal cultivation condition in autumn. SP represents squaring period. FT represents flowering time. DE represents 10 cm-high elongated floral stalk. Sigmaplot software (Systat software, CA, USA) was used for statistical analysis. **c** The distribution of SNP index in offspring pool on chromosome A04 generated by MutMap analysis. Sliding window was performed with a simple perl script using a window size of 1 Mb and a step size of 10 Kb as the default settings. **d** Sequencing peak of the C/T allele of SNP 13,129,878 generated by Sanger sequencing. The raw chromatogram data were analyzed using Chromas software

Following EMS treatment, 528 M_0_ lines were obtained. By continuous identification and further screening for generations, the mutant *ebm3* exhibiting obvious early-bolting characteristics in spring and autumn cultivation was selected as the study material. Except for curled leaves, the mutant *emb3* showed no other pleiotropic effects when compared with the wild-type line ‘FT’ (Fig. 1a).

Under normal cultivation conditions in autumn, the wild-type line ‘FT’ will not prematurely bolt without exposure to a prolonged cold period (vernalization); however, but the mutant *emb3* exhibited obvious bolting under these conditions (Fig. 1a). To more intuitively assess the characteristics of the mutant, three indices, squaring period (SP), flowering time (FT), and days to reaching a 10 cm-high elongated floral stalk (DE), were measured in 30 individuals and the average values are presented. SP, FD, and DE of the mutant *ebm3* were 40, 43, and 46 days, respectively (Fig. 1b).

The reciprocal cross F_1_ generation had the same phenotype as the wild-type line ‘FT’, indicating that the early-bolting phenotype of the mutant *ebm3* was recessive and controlled by nuclear gene. In the F_2_ generation, 1,225 and 401 individuals exhibited the wild-type line ‘FT’ and mutant *ebm3* phenotype, respectively. This segregation ratio was consistent with the Mendelian ratio of 3:1 segregation (χ^2^ = 0.08 < χ^2^_0.05_ = 3.84). In addition, all 518 BC_1_ (F_1_ × ‘FT’) generation individuals exhibited the phenotype of the wild-type line ‘FT’. For the BC_1_ (F_1_ × *ebm3*) generation, 264 and 272 individuals exhibited the wild-type line ‘FT’ and mutant *ebm3* phenotype, respectively. This 1:1 segregation ratio was consistent with the expectations (χ^2^ = 0.09 < χ^2^_0.05_ = 3.84). These data indicated that the phenotype of the mutant *ebm3* was controlled by a single, recessive nuclear gene, named *BrEBM3*, and its independent allele is *Brebm3* (Table 1).

**Table 1:** Genetic analysis of the early-bolting mutant *ebm3* of Chinese cabbage

Generation	‘FT’	*ebm3*	Total	Segregation Ratio	Expected Ratio	χ^2^
P_1_ (‘FT’)	92	0	92			
P_2_ (*ebm3*)	0	81	81			
F_1_ (P_1_×P_2_)	192	0	192			
F_1_ (P_2_×P_1_)	184	0	184			
BC_1_ (F_1_×‘FT’)	518	0	518			
BC_1_ (F_1_×*ebm3*)	264	272	536	0.97: 1	1:1	0.08
F_2_	1225	401	1626	3.05: 1	3:1	0.09

### Identification of the candidate gene of the mutant *ebm3*

We established three bulks to identify the bolting gene by a modified MutMap method [40]. The offspring pool comprised 15 plants exhibiting the mutant phenotype in the F_2_ population. The DNA of the offspring pool, wild-type ‘FT’ and mutant *ebm3* was used to construct three libraries, designed as F_2__ebm3, ‘FT’ and ebm3. The three libraries were resequenced. Sequencing produced 48.75 Gb of raw data. After filtering, 18.98 Gb, 9.19 Gb, and 20.55 Gb clean data were obtained for the ‘FT’, ebm3, and F_2__ebm3 library, respectively. Sufficient data were produced for each library, the sequencing data were of sufficient quality (Q20 ≥ 93.69 %, Q30 ≥ 88.04 %), and the GC distribution (37.55–42.77 %) was normal (Additional file 2: Table S1). The clean reads were aligned to the *B. rapa* reference genome (v3.0). The mapping rate was 97.95 %, 97.37 %, and 97.31 % for the ‘FT’, ebm3, and F_2__ebm3 library, respectively, corresponding to an average read coverage of 48.00, 22.46, and 21.17 X (Additional file 2: Table S2). The mapped reads were normal and could be used for subsequent SNP detection. In total, 414,354 SNPs were detected in the ‘FT’ and ebm3 library, of which 457 homozygous nonsynonymous loci were gained to calculate SNP index in F_2__ebm3 library. And then these loci that the SNP index < 0.3 were filtered out. To evaluate the distribution of the SNP index physically mapped across the *B. rapa* chromosomes, we used an sliding window method with a 1 Mb window size and 10 Kb step size in F_2__ebm3 library. Here, we only showed the distribution of the SNP index in offspring pool on Chromosome A04, because it was the chromosome where the candidate loci were located (Fig. 1c). To further confirm the candidate loci, these above SNPs were screened as follows: retain loci with SNP index = 1; filter out not-typical EMS mutant loci; retain loci large-effect. Finally, six SNPs (3,407,432, 6,258,734, 13,129,878, 18,591,168, 21,580,928 and 20,708,402) were identified on chromosome A04, including five nonsynonymous sites in exons and one alternative splice site in an intron (Table 2).

**Table 2:** List of the candidate SNPs identified by MutMap analysis

ID	Pos	Ref	Alt	SNP index	Variant	Description
*BraA04g005220.3 C*	3,407,432	G	A	1	nonsynonymous	CDT1-like protein b isoform X3
*BraA04g008870.3 C*	6,258,734	G	T	1	nonsynonymous	LOW QUALITY PROTEIN: short-chain type dehydrogenase/reductase
*BraA04g017190.3 C*	13,129,878	C	T	1	nonsynonymous	histone-lysine N-methyltransferase CLF isoform X1
*BraA04g026040.3 C*	18,591,168	C	T	1	nonsynonymous	LEA protein group 3
*BraA04g031990.3 C*	21,580,928	C	T	1	nonsynonymous	39 S ribosomal protein L46, mitochondrial-like
*BraA04g030150.3 C*	20,708,402	G	A	1	splicing	signal recognition particle 14 kDa protein-like

To verify the reliability of these six mutated SNPs, the sequences surrounding them were amplified from DNA from the mutant *ebm3* and wild-type line ‘FT’. Sequence alignment results showed that all SNPs were real and the sequencing peak of the C/T allele of SNP 13,129,878 was displayed in Fig. 1d.

We conducted genotyping analysis of 200 F_2_ individuals to confirm the candidate SNP for the early-bolting mutant phenotype. A KASP assay showed that SNP 13,129,878 of *BraA04g017190.3 C* co-segregated with the mutant phenotype in the F_2_ individuals. All F_2_ individuals exhibited a T:T genotype, whereas the wild-type line ‘FT’ was C:C genotype. For the other five SNPs, recombinants were detected in the F_2_ individuals, indicating these SNPs did not co-segregate with the mutant phenotype (Additional file 2: Table S3). These results confirmed that *BraA04g017190.3 C*, harboring SNP 13,129,878, was the candidate gene of the mutant *ebm3*. Gene annotation confirmed that *BraA04g017190.3 C* encoded an important histone methyltransferase, homologous to CLF. Loss-of-function of *A. thaliana CLF* (*At2g23380*) causes an early flowering phenotype and upwardly curled leaves [41]. In this study, the candidate gene of the mutant *ebm3* is referred to as *BrEBM3.*

**Fig. 2:**
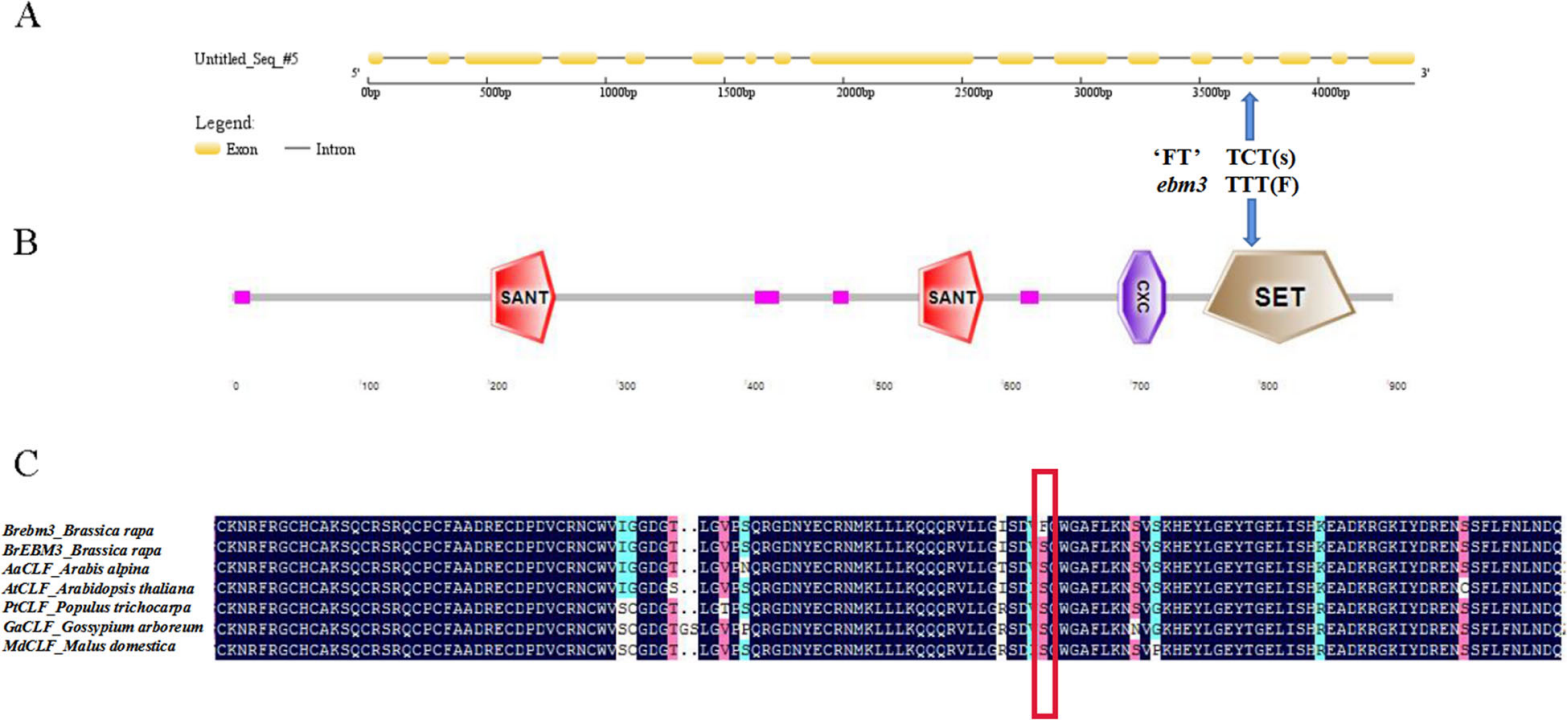
Sequence analysis of *BrEBM3.***a** Gene structure of *BrEBM3* and the site of nonsynonymous base substitution. **b** Conserved domain analysis of *BrEBM3* and the site of amino acid change. **c** Sequence alignment of CLF in various species. The CLF protein accession numbers are follows: *Arabis alpina*, AaCLF (KFK32747.1); *Arabidopsis thaliana*, AtCLF (CAA71599.1); *Populus trichocarpa*, PtCLF (XP_024460754.1); *Gossypium arboreum*, GaCLF (KHG06300.1); *Malus domestica*, MdCLF (XP_008340296.2)

The full-length gene sequence of *BrEBM3* was found to be 4,406 bp, and *BrEBM3* consists of 17 exons and 16 introns (Fig. 2a). Sequence alignment showed that besides SNP 13,129,878 in the 14th exon, there was no variation in the gene sequence and promoter sequence (2,000 bp upstream of the initiation codon) between the mutant *ebm3* and wild-type line ‘FT’. The coding sequence of *BrEBM3*, 2,715 bp in length, encodes a protein of 904 amino acids with a molecular weight of 1000 kDa and a theoretical pI of 90.5. The SNP 13,129,878 (C→T) of *BrEBM3* causes an amino acid substitution from serine (S) to phenylalanine (F) at residue 766 (Fig. 2b). The amino acid substitution is localized in a typical SET domain that is highly conserved among diverse species (Fig. 2c; The original figure refers to Additional file 1: Figure S1).

### Functional verification by allelic mutants

The mutant *ebm1* was screened from our early bolting mutant lines, presenting identical phenotype with *ebm3*. In mutant *ebm1*, *BraA04g017190.3 C* harbored a novel 53 bp insertion that caused the termination of amino acid coding, was previously predicted as a candidate mutant gene for early bolting in Chinese cabbage [42]. Our study revealed that mutant *ebm3* was caused by a C to T nonsynonymous mutation in the exon of *BraA04g017190.3 C*. To determine the allelism of mutant *ebm1* and *ebm3*, they were crossed with each other. The phenotype of the hybrid was the same as that of the two mutants, which suggested that mutant *ebm1* and *ebm3* were controlled by an allelic gene. Both allelic mutations of *BraA04g017190.3 C* in *ebm3* and *ebm1* conferred the similar early bolting phenotype, which reciprocally verified the *BrEBM3* function in two allelic mutants.

### Spatiotemporal expression of *BrEBM3*

**Fig. 3:**
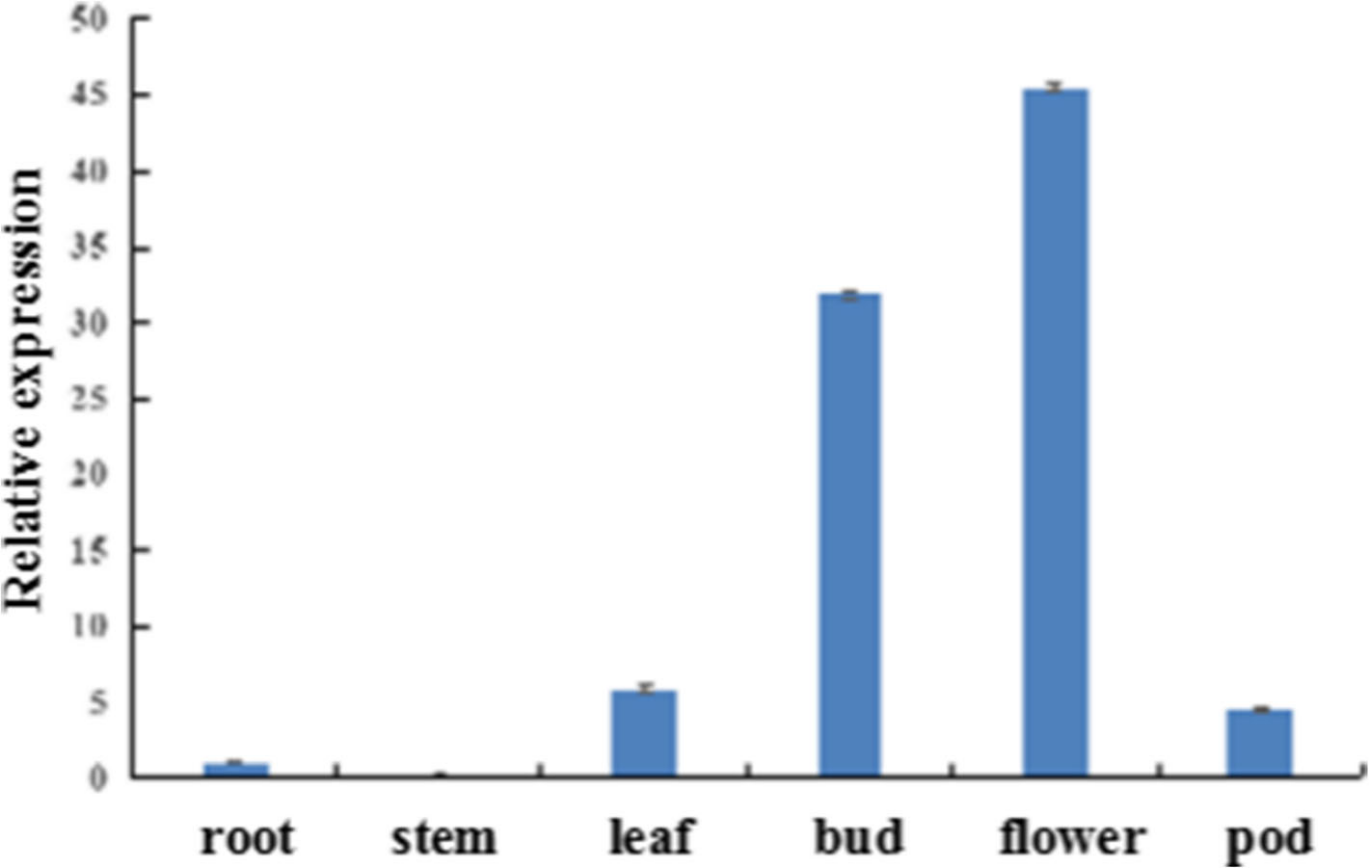
Spatiotemporal expression of *BrEBM3* in different tissues of the wild-type line ‘FT’

To study the relative expression levels of *BrEBM3* in different tissues, RNA from root, stem, leaf, bud, flower, and pod of the wild-type line ‘FT’ was used as a template for qRT-PCR. The data showed that *BrEBM3*expression was the highest in the flower, followed by the bud, leaf, and pod, with extremely low expression in the stem (Fig. 3).

### *BrEBM3* promoter activity

**Fig. 4:**
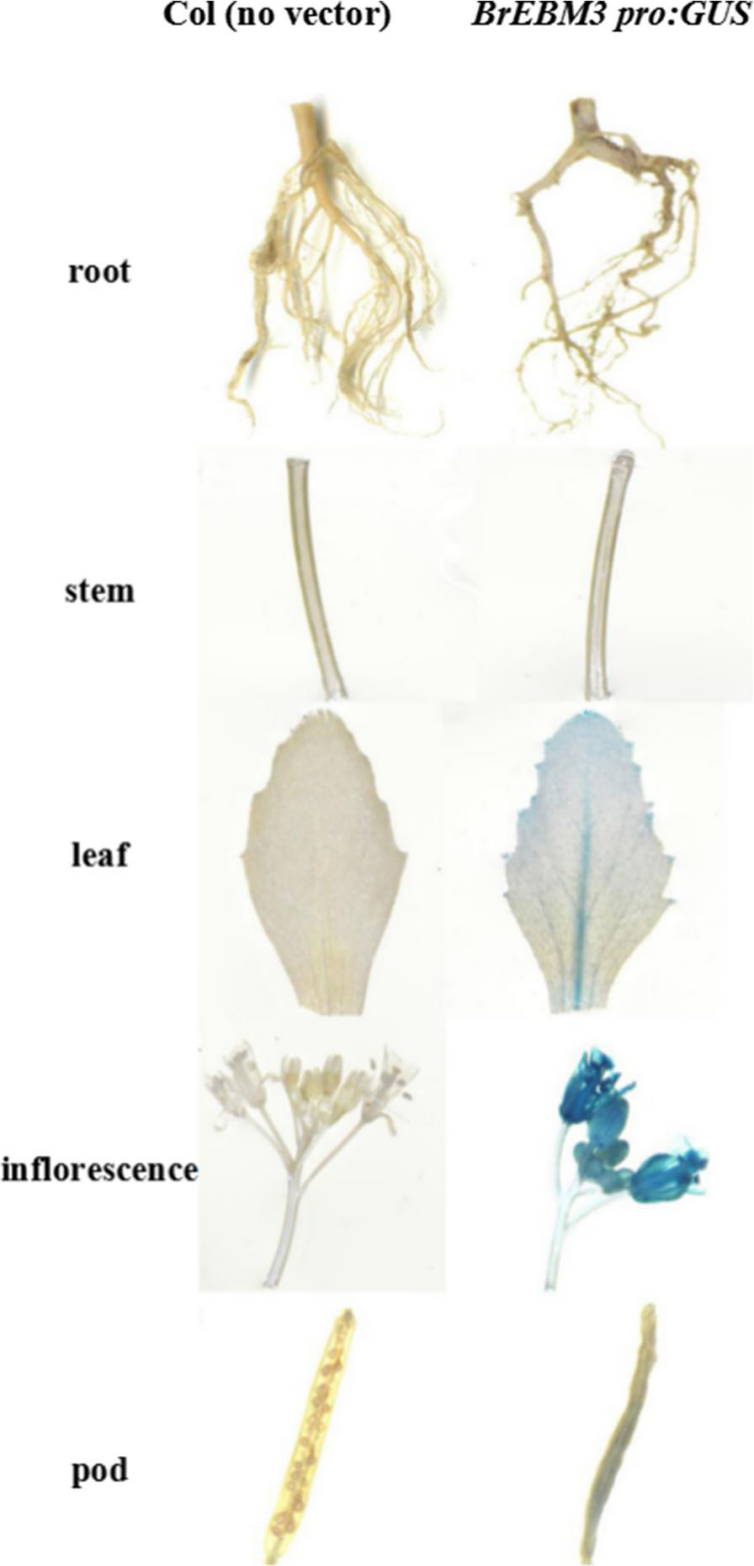
GUS staining of different tissues in homozygous T_2_-generation transgenic plants expressing the GUS gene under control of the 2000 bp promoter sequence of *BrEBM3*. The tissues were examined under Eclipse 80i microscope (Nikon Corporation, Japan)

According to MutMap and cloning sequencing, the promoter sequence of *BrEBM3* was not different between the mutant *ebm3* and wild-type line ‘FT’. Therefore, we just analyzed *BrEBM3* promoter activity in *A. thaliana* tissues by using the fusion vector *BrEBM3 pro:GUS.* Following screening based on hygromycin resistance and the *GUS* reporter gene, 32 independent transgenic plants were obtained. Tissues (root, stem, leaf, inflorescence, and pod) of homozygous T_2_ generation transgenic plants were stained in a GUS histochemical assay. Analysis of the transformed plants showed that *BrEBM3* transcriptional activity was the highest in the inflorescence, followed by leaf and pod (Fig. 4). These results were in line with those of spatiotemporal expression analysis, indicating that *BrEBM3* expression shows a tissue-specific pattern.

### BrEBM3 is located to the nucleus

**Fig. 5:**
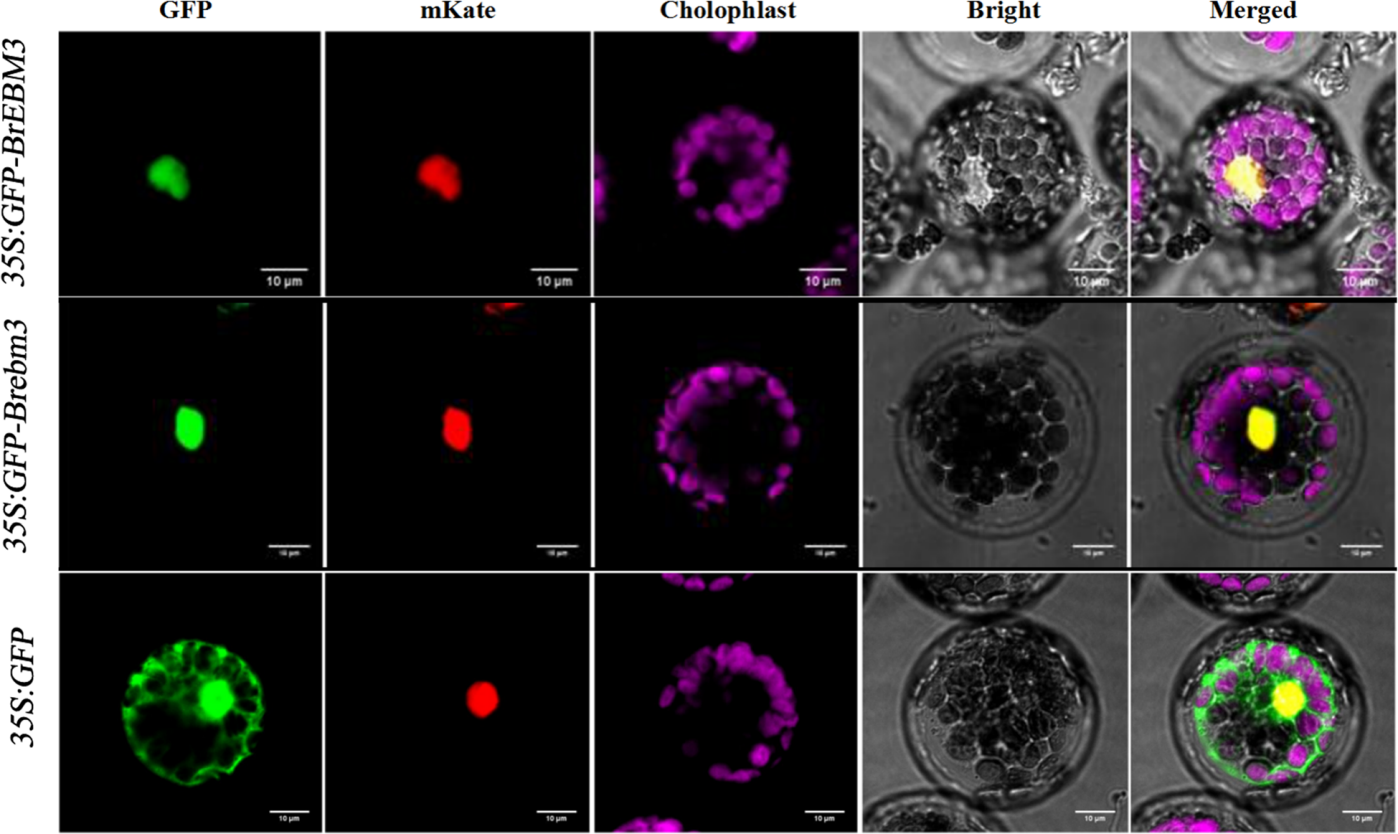
Subcellular localization analysis of BrEBM3. The *35 S:GFP-BrEBM3*, *35 S:GFP-Brebm3* and blank *35 S:GFP* vectors were transiently expressed in *Arabidopsis* mesophyll cell protoplasts. The nuclear localization signal (NLS) protein fused with mKate was used as a nucleus marker. Confocal images were captured 24-48 h after inoculation and observed under a confocal laser-scanning microscope. The merged images include GFP channel (green), mKate channel (red), chloroplast autofluorescence channel (pink) and bright field. Scale bar, 10 μm

To detect the subcellular localization of BrEBM3, we constructed recombinant *35 S:GFP-BrEBM3* vector for transiently expression. Co-localization analysis of GFP and mKate fluorescent signals in the transformed *Arabidopsis* mesophyll cell protoplasts indicated that the fusion protein was exclusively located in the nucleus, suggesting that BrEBM3 is a nucleoprotein. And the 3*5 S:GFP* control vector was detected within both the nucleus and cytoplasm. To prove that BrEBM3 functions at the site of the nucleus, we also expressed the *35 S:GFP-Brebm3* vector in *Arabidopsis* mesophyll cell protoplasts. Again, the fluorescent signal was strong in the nucleus. Thus, BrEBM3 is located to the nucleus (Fig. 5).

### Transcriptome profiling of the mutant *ebm3*

To analyze the molecular mechanism of *BrEBM3* in regulating early bolting in Chinese cabbage, a comparative transcriptome analysis of the SAM of mutant *ebm3* and wild-type line ‘FT’ was conducted with three biological replication. After filtering and quality control, 22.32 Gb and 20.94 Gb of clean reads were obtained from the ‘FT’ and ebm3 library, respectively. Sufficient data were produced for each library, the sequencing quality (Q20 ≥ 99.91 %, Q30 ≥ 98.41 %) was sufficient, and the GC distribution (46.50–47 %) was normal (Additional file 2: Table S4). On average, 69.01 and 68.93 % of clean reads from ‘FT’ and ebm3 libraries, respectively, were uniquely mapped to the *B. rapa* reference genome (v3.0) (Additional file 2: Table S5). In total, 1,906 DEGs, including 1,079 up- and 827 downregulated genes, were identified in the mutant *ebm3* (Additional file 2: Table S6). Of the DEGs, were specifically expressed, with 81 and 79 specifically expressed in mutant *ebm3* and wild-type line ‘FT’, respectively (Additional file 2: Table S7). To determine their biological functions, we used GO term and KEGG pathway enrichment analysis (Additional file 1: Figure S2 and Figure S3). We identified 272 significantly enriched GO terms (*p* vaule ≤ 0.03) (Additional file 2: Table S8). Of these, 163, 13, and 96 GO terms were in the biological process, cellular component and molecular function, respectively. The most significantly enriched GO terms were “regulation of transcription, DNA-templated” (GO:0006355; 193 DEGs) in biological process, “plasma membrane” (GO:0005886; 329 DEGs) in cellular component, and “transcription factor activity, sequence-specific DNA binding” (GO:0003700; 184) in molecular function. We identified 19 significantly enriched KEGG pathways (*p* vaule ≤ 0.03) (Additional file 2: Table S8). Of these, starch and sucrose metabolism (ko00500; 76), phenylalanine metabolism (ko00940; 42), and circadian rhythm-plant (ko04712; 39) were the most significantly enriched metabolic pathway.

**Fig. 6:**
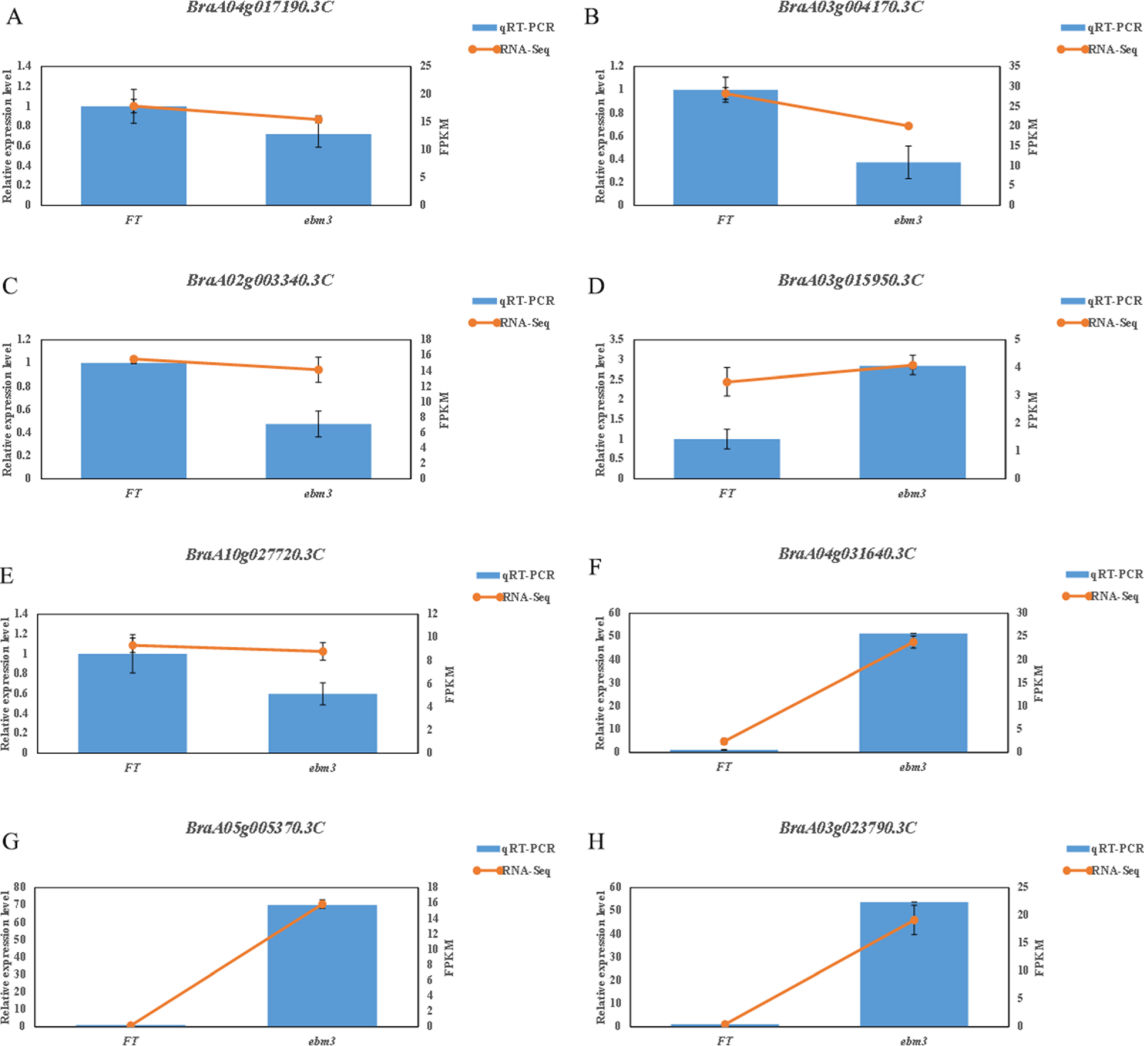
Expression analysis of genes associated with flowering time regulation in the SAM of the mutant *ebm3* and wild-type line ‘FT’. **a** Analysis of *BrEBM3* expression; **b-e** Analysis of *FLC* expression. **f-h** Analysis of *SOC1* expression. Sigmaplot software was used for statistical analysis

Flowering is an essential stage in the life cycle of higher plants and is tightly controlled by complex molecular pathways. To further explore the molecular mechanism underlying the early-bolting phenotype of the mutant *ebm3*, we conducted an in-depth analysis of the transcriptome data. The candidate gene *BrEBM3* (*BraA04g017190.3 C*) was not significantly differentially expressed between the mutant *ebm3* and wild-type line‘FT’ (Additional file 2: Table S10; Fig. 6a). The floral integrator genes *FT*, *TSF*, *TFL1*, and *SOC1*, the vernalization pathway-related genes *FLC* and *FRI*, the ambient temperature-related gene *SVP*, the photoperiod pathway-related gene *GI*, age pathway-involved genes *SPL3*, *SPL9* and *SPL15*, gibberellin pathway-involved genes *GA20OX1*-*4*, floral homeotic genes *AG*, *AGL19*, and *FUL*/*AGL8* were searched in our data. CLF-repressed genes (CRGs), including *SOC1* genes (*BraA04g031640.3 C*, *BraA05g005370.3 C* and *BraA03g023790.3 C*), two *AG* genes (*BraA03g048590.3 C* and *BraA01g010430.3 C*), *AGL19* (*BraA01g013570.3 C*) were significantly upregulated in the mutant *ebm3* as compared to wild-type line ‘FT’ (Additional file 2: Table S10). We assessed *BrEBM3* (*BraA04g017190.3 C*), *FLC* (*BraA02g003340.3 C*, *BraA03g004170.3 C*, *BraA03g015950.3 C* and *BraA10g027720.3 C*), and *SOC1* (*BraA04g031640.3 C*, *BraA05g005370.3 C* and *BraA03g023790.3 C*) expression by qRT-PCR to verify the reliability of the RNA-seq data. As described by RNA-Seq, the cDNAs from the SAM of mutant *ebm3* and wild-type line ‘FT’ were collected with three biological replicates, respectively. The six new samples were designated as emb3-4, emb3-5, emb3-6, ‘FT’-4, ‘FT’-5, and ‘FT’-6. As shown in Fig. 6, the expression patterns of the eight genes were generally consistent with the RNA-seq data, indicating the reliability our transcriptome analysis.

## Discussion

A good understanding of the molecular mechanism of flowering time can accelerate the breeding of bolting-resistant varieties [29]. To adapt to the diverse agro-environments, vegetable crops have employed a complex and elaborate network that tightly controls flowering time. Mutants are important materials for plant functional genomics studies. The genetic basis of natural variation in flowering time has been extensively evaluated in quantitative trait loci (QTL) studies [43–46]. However, there is insufficient natural variation for effective research due to the low probability. Here, we characterized an EMS-induced early-bolting mutant, *ebm3*, with curled leaves, which was derived from a Chinese cabbage DH line ‘FT’ (Fig. 1a). The genetic background of the mutant *ebm3* was relatively homozygous, and highly consistent with that of the wild-type line ‘FT’, which was conducive to highlight the bolting phenotype caused by the causal gene. Genetic analysis showed that the mutant trait was quality character, controlled by a single recessive nuclear gene (Table 1). Multi-season planting indicated that the early-bolting trait was genetically stable and not affected by external factors. Therefore, the mutant *ebm3* is an ideal material to study important node genes in the flowering regulatory pathways in Chinese cabbage.

EMS mutagenesis has multiple advantages, such as high mutation frequency, easy screening, and stable inheritance, which is why EMS is the most widely used chemical mutagen in plants [47]. The combination of high-throughput sequencing with bulk segregant analysis (BSA) has laid the foundation for rapid mining of new genes using mutants, which has greatly facilitated functional genome studies. In our study, we used a modified MutMap method and KASP genotyping to map the candidate gene. *BraA04g017190.3 C* encoding histone methyltransferases CLF, was found to be responsible for the early-bolting trait (Fig. 1c-d). A nonsynonymous SNP in the 14th exon of *BraA04g017190.3 C* caused an amino acid substitution from S to F (Fig. 2a). Unlike loss-of-function of *Arabidopsis CLF*, a single amino acid change of in the Enhancer of zeste (E(z)) ortholog *CLF*, *clf-59* retained *FLC* repression by promoting histone H3 lysine 27 trimethylation (H3K27me3) deposition in *FLC* chromatin, causing early flowering [48]. Sequence comparison of CLF of various species revealed that the protein has a highly conserved SET domain, and the nonsynonymous SNP was located in this domain (Fig. 2b, c). The SET domain is a 130–140-amino acid evolutionarily conserved sequence motif [49]. SET domain proteins have been characterized in diverse plant species, including *Arabidopsis*, rice, maize, barley, grapevine, and poplar [50–55]. Most histone lysine methyltransferases (HKMTases) have a conserved SET domain, and the HKMTases with SET domain are named SET DOMAIN GROUP (SDG) proteins. Plant SDG proteins are generally divided into four classes: suppressor of protein-effect variegation 3–9 (SU(VAR)3–9); E(z); trithorax (TRX); and absent, small or homeotic disks 1 (ASH1). Detrimental effects of mutations in E(z), TRX, and ASH1 mark the importance of the SET domain in plant growth and development [56]. Moreover, SDG proteins have been suggested to affect flowering time. Mutations in five *Arabidopsis* SDG genes, including *ASHR3*/*SDG2* [57], *ASHH2*/*SDG8*/*EFS* [58], *ATX1*/*SDG27* [59], *ATXR7*/*SDG25* [60], and *CLF*/*SDG1* [41], cause an early-flowering phenotype, and mutations in *Arabidopsis ASHH1*/*SDG26* [61], and three rice genes, including *SDG708* [62], *SDG724* [63] and *SDG725* [64], confer a late-flowering phenotype. In summary, there is strong evidence that an amino acid residue substitution in the SET domain of BraA04g017190.3 C leads to the early-bolting phenotype in Chinese cabbage. Since we do not do any rescue or complementation, we can’t rule out other possibilities, such as epigenetics, non-coding sequences, etc. But more importantly, allelic mutant *ebm1* was screened from our early bolting mutant lines, presenting identical phenotype with *ebm3*. To determine the allelism of mutant *ebm1* and *ebm3*, they were crossed with each other. The phenotype of the hybrid was the same as that of the two mutants, which suggested that mutant *ebm1* and *ebm3* were controlled by an allelic gene. Our study revealed that mutant *ebm3* was caused by a C to T nonsynonymous mutation in the exon of *BraA04g017190.3 C*. Unlike that in *ebm*3, the *BraA04g017190.3 C* in *ebm1* had a novel 53 bp insertion that caused the termination of amino acid coding. As stated above, our data revealed that *BraA04g017190.3 C*. is related to bolting in Chinese cabbage.

Epigenetic factors play crucial roles in flowering regulation by activating or repressing the transcription of flowering genes. Two functionally distinct multiprotein complexes of the Polycomb Group (PcG), PcG Repressive Complex 1 (PRC1) and PRC2, are the core epigenetic factors in eukaryotes [65]. PRC2 is a key repressive epigenetic mark, which maintains the repressed state of a target gene by catalyzing H3K27me3 [66]. In *A. thaliana*, PRC2 acts on various growth and developmental processes, including leaf morphology, floral organogenesis, cell pluripotency, vegetative-to-reproductive phase transition, and embryonic development [67–72]. In *A. thaliana*, CLF is the main component of the E(z) subunit of PRC2 [71]. Extensive evidence supports that CLF maintains suppressed expression of *FLC* and *FT*, as well as that of several floral homeotic genes, including *AG*, *AGL19*, and *SEP3* [41, 71, 73–77]. As a typical example of reprogramming of epigenetic states in plants, H3K27me3 repressive marks on *FLC* can be erased by ELF6 histone demethylases during seed development [78]. A noncoding RNA transcribed from the second intron of *AG* associated with CLF can silence *AG* expression by mediating H3K27m3 deposition to form repressive chromatin [79]. The temporal-specific interaction of NF-YC and CLF mediates epigenetic regulation by derepressing *FT* expression in photoperiod-induced flowering [6]. Loss-of-function of *ASHH1/SDG26* retains *SOC1*/*AGL20* repression by reducing H3K4me3 and H3K36me3 deposition in *SOC1*/*AGL20* chromatin, resulting in the late-flowering phenotype [61]. A tilling mutant of *(B) rapa*, braA.clf-1 (Gln615Stop), displayed small plant size, altered floral development, and curled leaves due to reduced H3K27me3 and high expression levels of floral homeotic genes such as *AG* and *AGL* loci [80]. In the present study, RNA-Seq data revealed that *AG* and *AGL* loci, e.g., *SOC1*/*AGL20* and *AGL19*, were significantly upregulated in the mutant *ebm3* (Additional file 2: Table S10). Therefore, it is reasonable to speculate that mutation in *BrEBM3* mediates reduced H2K27me3 deposition and high expression of the *AG* and *AGL* loci in Chinese cabbage. Hereafter, to determined significant enrichment of H3K27me3 epigenetic marks at genome-wide levels, chromatin immunoprecipitation, followed by sequencing (ChIP-seq) assays should be developed from the SAM of the mutant *ebm3*. The data from ChIP-seq can strengthen the information generated from our RNA-Seq to H3K27me3 levels at CRGs, e.g., *AG* and *AGL* loci. In addition, the H3K27me3 levels of the CRGs should be verified by locus-specific PCR on ChIP-derived DNA-materials (ChIP-PCR) [81].

## Conclusions

The transition to flowering is an essential developmental stage in the plant life cycle. Plants need to flowering in the most favorable conditions to ensure maximal reproductive success. Timely flowering is conducive to crop production, harvesting, and marketing. In this study, based on MutMap sequencing, KASP genotyping, and allelism test, *BrEBM3*, encoding the histone methyltransferase CLF, was determined to control the early-bolting trait in Chinese cabbage. *BrEBM3* was highly expressed in the floral organs, and the translation product localized in the nucleus. Transcriptome profiling was conducted to identify potential CLF-repressed genes in mutant *ebm3*. Collectively, our findings will be invaluable for understanding the molecular mechanism of flowering time in Chinese cabbage.

## Methods

### Plant materials

The Chinese cabbage DH line ‘FT’ was used as a wild-type line in this study propagated from Chinese cabbage variety ‘Fukuda 50’, which was screened by Shenyang greenstar Chinese cabbage research institute (Shenyang, China) [82]. An early-bolting mutant with stable inheritance was obtained from ‘FT’ seeds by multigenerational screening after EMS mutagenesis, and was designated *ebm3*. The mutant generation method has been described in detail in Fu et al. [83]. All plants were raised in the greenhouse of Shenyang Agricultural University.

A. *thaliana* ecotype Columbia-0 (Col-0) was obtained from the Arabidopsis Biological Resource Center (ABRC; http://abrc.osu.edu) and preserved by the Liaoning Key Laboratory of Genetics and Breeding for Cruciferous Vegetable Crops at Shenyang Agricultural University. All Arabidopsis plants were grown in a growth chamber at Shenyang Agricultural University. Culture conditions were as described by Wang et al. [84].

### Genetic analysis

To study the inheritance characteristics, the mutant *ebm3* and wild-type line ‘FT’ were used as parents. An F_1_ generation obtained by a reciprocal cross was self-crossed to obtain an F_2_ segregating generation. The F_1_ generation was backcrossed with both parents to obtain a BC_1_ population. The segregation ratios of the F_2_ and BC_1_ populations were analyzed using the chi-square test. The F_2_ population was also used for mutant gene identification and genotyping. Individual plants were grown in a greenhouse at Shenyang Agricultural University.

### Allelism test between the *ebm1* and *ebm3* mutants

A early bolting mutant *ebm1* presenting identical phenotypes, was likewise derived from EMS mutagenesis of ‘FT’ seeds. To determine the early bolting traits of the two mutants from the allelic gene mutation, the mutants were reciprocally crossed.

### Evaluation of bolting characteristics

Three bolting characteristics were measured, i.e., SP, DE, and FT, as previously reported by Yu et al. [85]. Thirty mutant *ebm3* plants (10 individuals per replication, with three replicates each) were selected for a survey of bolting characteristics in comparison with wild-type line ‘FT’ under natural conditions in the autumn of 2017.

### Candidate SNP identification by the MutMap method

A modified MutMap method was used to identify the candidate gene for the mutant *ebm3*. DNA was extracted from 15 F_2_ individuals with the early-bolting phenotype and the parental lines using a DNAsecure Plant Kit (Tiangen Biotech Co., Ltd., Beijing, China) according to the manufacturer’s instructions. Equal amounts of each DNA from the 15 F_2_ individuals were mixed to construct an offspring pool. Sequencing libraries of the mutant *ebm3* (ebm3), wild-type line ‘FT’ (‘FT’), and offspring pool (F_2__ebm3) were generated using a TruSeq Nano DNA HT Sample preparation Kit (Illumina, San Diego, CA, USA). The libraries were sequenced using Illumina HiSeq^TM^PE150 (Novogene Co., Ltd., Beijing, China). After quality control and filtration, the clean reads of each sample were aligned to the *B. rapa* reference genome (http://brassicadb.org/brad/, v3.0) using Burrows-Wheeler Alignment tool (BWA) [86]. Alignment files were converted to BAM files using the SAMtools software [87]. SNP calling was performed using GATK [88] and annotated using ANNOVAR [89]. The screened SNPs between the M and W library were used to calculate the SNP index in offspring-pool library. The sliding window method was used to determine the SNP index of the whole genome in offspring pool library.

### SNP genotyping by KASP

To verify the real existence of the candidate SNP, a sequence surrounding the locus was amplified using DNA from the mutant *ebm3* and wild-type line ‘FT’ and the primer pair 5′-ATACTTTGCTTTGGTTGACTCTAC-3′ and 5′-TCGTGTTTACTTACACTGTTCTGT-3′. Purified PCR product was ligated into the PMD 18-T Vector (Takara Biotech Co., Ltd., Dalian, China), and transformed into TOP10 competent cells (ComWin Biotech Co., Ltd., Beijing, China). The recombinant plasmid was sequenced by Sanger sequencing (Genewiz lnc., Tianjin, China). Sequence alignment was performed using the SeqMan software.

The candidate SNP was confirmed using a KASP assay to detect whether the locus co-segregated with the mutant phenotype. For KASP genotyping, DNA from 200 F_2_ individuals with the early-bolting phenotype was used. Two allele-specific primers carrying the fluorescence probes FAM and HEX and the candidate SNP at the 3′ end (Primer_AlleleFAM: AGGTTTTACTTGGAATATCTGATGTATC; Primer_AlleleHEX: CAGGTTTTACTTGGAATATCTGATGTATT), and a common genome-specific primer (Primer_Common: GTTACGCATCTACTATACCTTTAGGAAAG), were designed following standard KASP guidelines of the laboratory of the Government Chemist (LGC http://www.lgcgenomics.com/). The primer mixture was prepared as recommended by LGC Genomics. PCR mixture preparation and cycling were conducted as described by Xi et al. [34]. Fluorescence data were read using a 7900HT Fast Real-Time PCR System (Applied Biosystems, Foster City, CA, USA).

### Quantitative reverse transcription-PCR (qRT-PCR)

Total RNA of each sample was extracted using TRIzol Reagent (Invitrogen, Carlsbad, CA, USA). First-strand cDNA was synthesized using FastKing gDNA Dispelling RT SuperMix (Tiangen Biotech Co., Ltd., Beijing, China). The reaction system was performed with UltraSYBR Mixture (ComWin Biotech Co., Ltd., Beijing, China). PCR amplification was run in a QuantStudio™ 6 Flex Real-Time PCR System (Applied Biosystems, Carlsbad, CA, USA). The *Actin* gene was selected as an internal control. Relative gene expression data were calculated by the 2^−△△Ct^ method [90]. The data were analyzed using the QuantStudio™ 6 Flex Manager software. Three technical and biological replicates were included for each sample. The qRT-PCR primer pairs were listed in Additional file 2: Table S11.

### Promoter activity assay

The promoter sequence (2,000 bp upstream of the initiation codon) of *BrEBM3* was amplified from DNA of the wild-type line ‘FT’, using the primer pair 5′-ccgggatccTCTAGAgcgaagccaagtagtaagcact-3′ and 5′-gcaggtcgacTCTAGAtgtcgaggagccagatcgga-3′ (uppercase letters indicate an *Xba*I site). The amplification product was digested with *Xba*I and ligated into the pC1301IgT vector containing fused GUS reporter gene. The recombinant plasmid was introduced into *Agrobacterium tumefaciens* strain GV3103. *A. tumefaciens*-mediated transformation was used to transfer the *BrEBM3 pro:GUS* vector into *A. thaliana* Col-0 by the floral dip method. Transgenic plants were screened on 0.5× Murashige and Skoog (MS) medium containing 0.25 mg L^− 1^ hygromycin. The GUS reporter gene was amplified from DNA of all hygromycin-resistant plants, using the primer pair 5′- AACCACAAACCGTTCTACTTTACTG-3′ and 5′-TACATTACAAGACGCTGCGAGT-3′. A GUS histochemical assay was performed on various tissues (root, stem, leaf, inflorescence and pod) of the transgenic plants [91].

### Subcellular localization

The full-length *BrEBM3* and its allele *Brebm3* coding sequence without the stop codon was amplified from cDNA of the wild-type line ‘FT’, using the primer pair 5′- cgatCACCTGCaaaacaacatggcgtcgggagcttcgcc-3′ and 5′-cagtCACCTGCaaaatacaagcaaccttcttgggtctac-3′ (uppercase letters indicate an *Aar*I site). The amplification product was digested with *Aat*I and inserted into the pBWA(V)HS-ccdb-GLosgfp vector, resulting in an N-terminal fusion vector with GFP under the control of the CaMV35S promoter (*35 S:GFP-BrEBM3 and 35 S:GFP-Brebm3*). The *35 S:GFP* vector was used as a control. The constructs were respectively transiently transformed into *A. thaliana* mesophyll cell protoplasts, as described by Wang et al. [84]. The pBWA(V)HS-NLS-mKATE vector was served as a nucleus marker. Fluorescence data were obtained by confocal laser-scanning microscope (Leica TCS SP8, Wetzlar, Germany).Excitation wavelengths used were 488nm for GFP and 561nm for mKate. Emission wavelengths were 507nm for GFP and 580nm for mKate.

### Transcriptome profiling

When the mutant *ebm3* reached the critical point of bolting, the SAM of three mutant and three wild-type line ‘FT’ was randomly selected and mixed; the mixed samples were used as one biological replicates. Three independent biological replicates of mutant and wild-type line ‘FT’ were used, respectively. Total RNA of the six samples (emb3-1, emb3-2, emb3-3, ‘FT’-1, ‘FT’-2, and ‘FT’-3) was extracted using TRIzol Reagent (Invitrogen, Carlsbad, CA, USA). RNA quantity and purity was analyzed using a Bioanalyzer 2100 and RNA 6000 Nano LabChip Kit (Agilent Technologies, Santa Additional file 2: Clara, CA, USA). Following purification and fragmentation, the cleaved RNA fragments were reverse-transcribed to create cDNA libraries using a mRNASeqsample preparation kit (Illumina, San Diego, CA, USA). The libraries were paired-end sequenced using an Illumina HiSeq 4000 platform (LC-Bio Technology Co., Ltd., Hangzhou, China). Following quality control and filtration, the clean reads were aligned to the *B. rapa* reference genome (v3.0) using HISAT. StringTie was used to assemble the alignments into transcripts and to compute transcript abundance by calculating Fragments Per Kilobase of transcript per Million mapped reads (FPKM). Differentially expressed genes (DEGs) were defined based on |log_2_(fold change)| ≥ 1 and *p* < 0.05, using the R package Ballgown [92]. Functional analysis of the DEGs included Gene Ontology (GO) and Kyoto Encyclopedia of Genes and Genomes (KEGG) pathway analyses [93, 94].

### Sequence characteristic analyses

The gene structure was displayed using the Gene Structure Display Server (http://gsds.cbi.pku.edu.cn/). Physical and chemical characteristics were predicted using Protparam (http://web.expasy.org/protparam). Domains were identified and annotated using Simple Modular Architecture Research Tool (http://smart.embl-heidelberg.de/).

## Supplementary Information

**Additional file 1: Figure S1.** Original figure of CLF sequence alignment in various species. The red line inner part is the cropping part in Fig. 2C. **Figure S2.** GO enrichment analysis of DEGs obtained in the SAM of mutant *ebm3* and wild-type line ‘FT’ by transcriptome profiling. **Figure S3.** Pathway enrichment analysis of DEGs obtained in the SAM of mutant *ebm3* and wild-type line ‘FT’ by transcriptome profiling.

**Additional file 2: Table S1.** Summary of sequencing data quality by MutMap analysis. **Table S2.** Summary of sequencing depth and coverage statistics by MutMap analysis. **Table S3.** Genotyping results of the six SNPs (3,407,432, 6,258,734, 13,129,878, 18,591,168, 20,708,40 and 21,580,928). **Table S4.** Summary of sequencing data quality by transcriptome profiling. **Table S5.** Summary of read statistics by transcriptome profiling. **Table S6.** List of 1,906 DEGs identified by transcriptome profiling. **Table S7.** List of specifically expressed genes identified by transcriptome profiling. **Table S8.** List of significantly enriched GO terms identified by transcriptome profiling. **Table S9.** List of significantly enriched KEGG metabolic pathways. **Table S10.** Information on genes associated with flowering time regulation identified by transcriptome profiling. **Table S11.** Primers used for qRT-PCR.

## Data Availability

The datasets supporting the conclusions of this article are included within the article and its additional files. The Illumina RNA-Seq datasets are available in the Sequence Read Archives (SRA) of the National Center for Biotechnology Information (NCBI) under the accession number SRR15152892, SRR15152891, SRR15152890, SRR15152889, SRR15152888, SRR15152887 of the Bioproject ID PRJNA746103. The DNA resequencing datasets are available in the SRA under accession number SRR15174648, SRR15174649, SRR15174650 of the Bioproject ID PRJNA746415. Genomic sequences and gene annotation information of *B.rapa* are downloaded online at http://brassicadb.cn.

## Abbreviations

CLF: CURLF LEAF
DEG: Differentially expressed genes
DH: Doubled haploid
EMS: Ethyl methanesulgonate
FPKM: Fragments Per Kilobase of transcript per Million mapped reads
GO: Gene Ontology
KASP: Kompetitive allele-specific polymerase chain reaction
KEGG: Kyoto Encyclopedia of Genes and Genomes
qRT-PCR: Quantitative reverse transcription-PCR
SAM: Shoot apical meristem
SET: Suppressor of protein-effect variegation 3-9, Enhancer-of-zeste, Trithorax
SNP: Single-nucleotide polymorphism
WGT: Whole genome triplication
