# Supplementary material for: A single amino acid residue substitution in BraA04g017190.3C, a histone methyltransferase, results in premature bolting in Chinese cabbage (Brassica rapa L. ssp. Pekinensis)
Source: BMC Plant Biol. 2021 Aug 13;21:373. doi: 10.1186/s12870-021-03153-9 (PMC8361648; doi:10.1186/s12870-021-03153-9)
Supplement: Supplementary file 1 — Additional file 1: Figure S1. Original figure of CLF sequence alignment in various species. The red line inner part is the cropping part in Fig. 2C. Figure S2. GO enrichment analysis of DEGs obtained in the SAM of mutant ebm3 and wild-type line ‘FT’ by transcriptome profiling. Figure S3. Pathway enrichment analysis of DEGs obtained in the SAM of mutant ebm3 and wild-type line ‘FT’ by transcriptome profiling. [file 12870_2021_3153_MOESM1_ESM.docx]

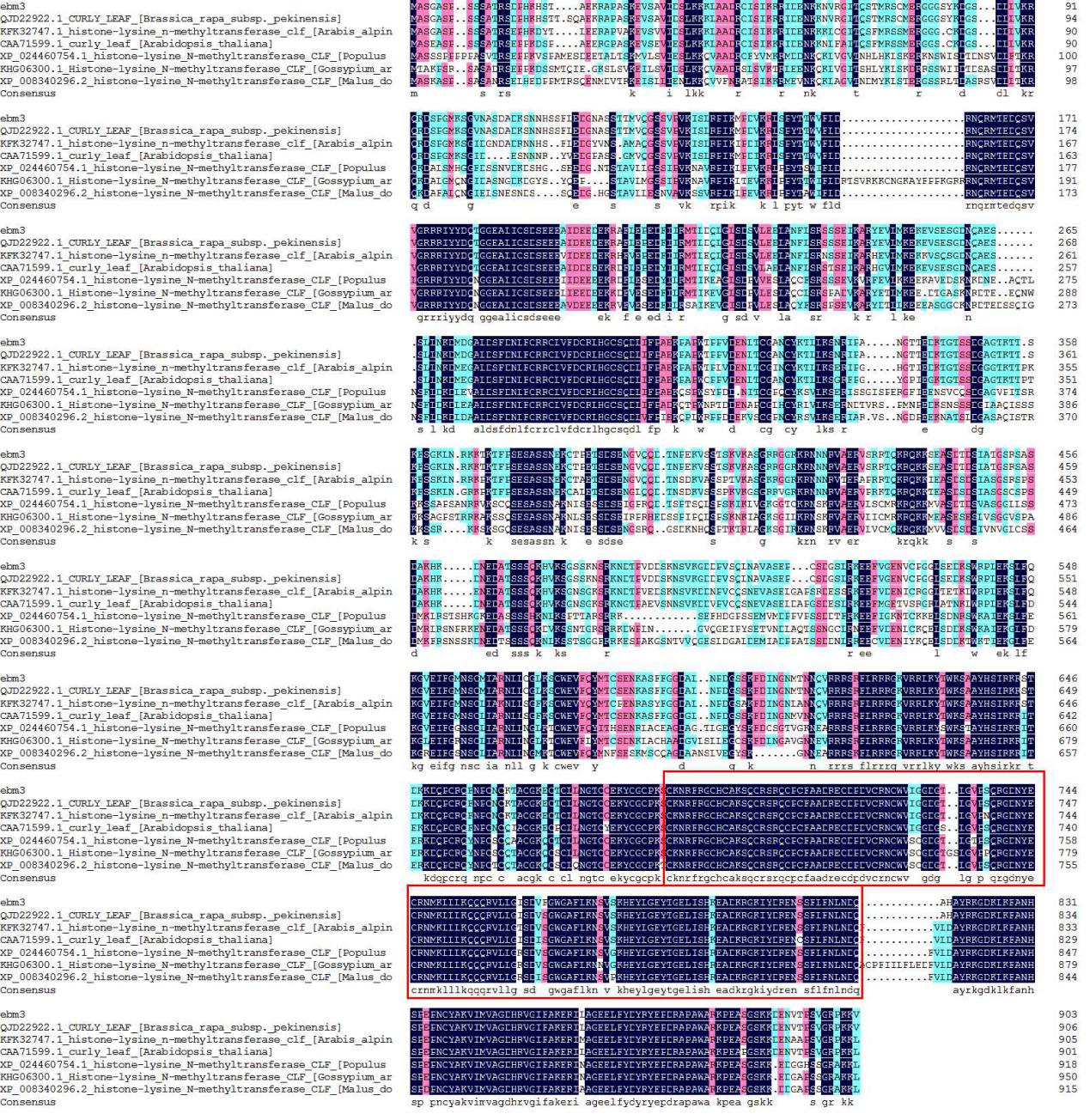


**Figure S1** Original figure of CLF sequence alignment in various species. The red line inner part is the cropping part in Fig. 2C.


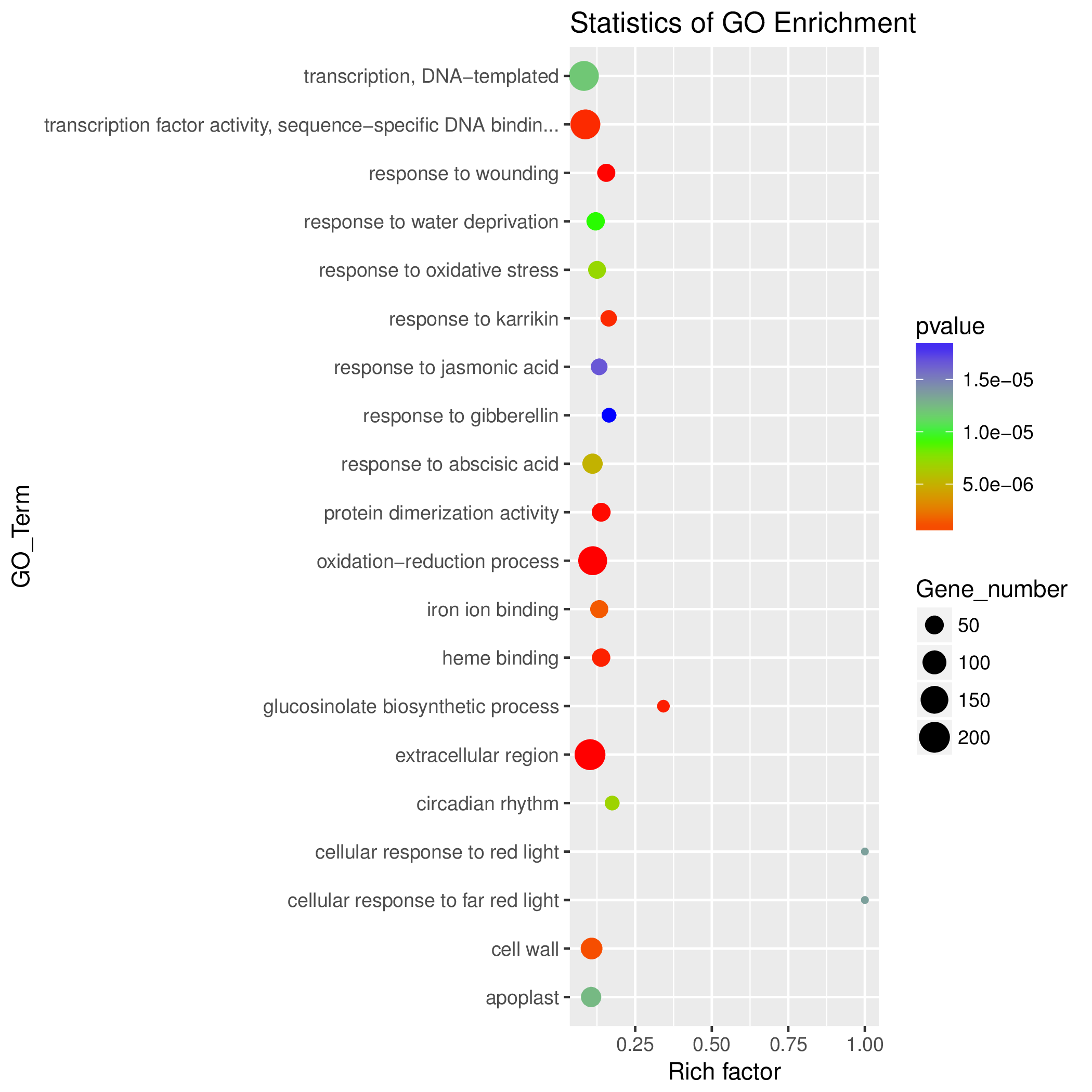


**Figure S2** GO enrichment analysis of DEGs obtained in the SAM of mutant ebm3 and wild-type line ‘FT’ by transcriptome profiling


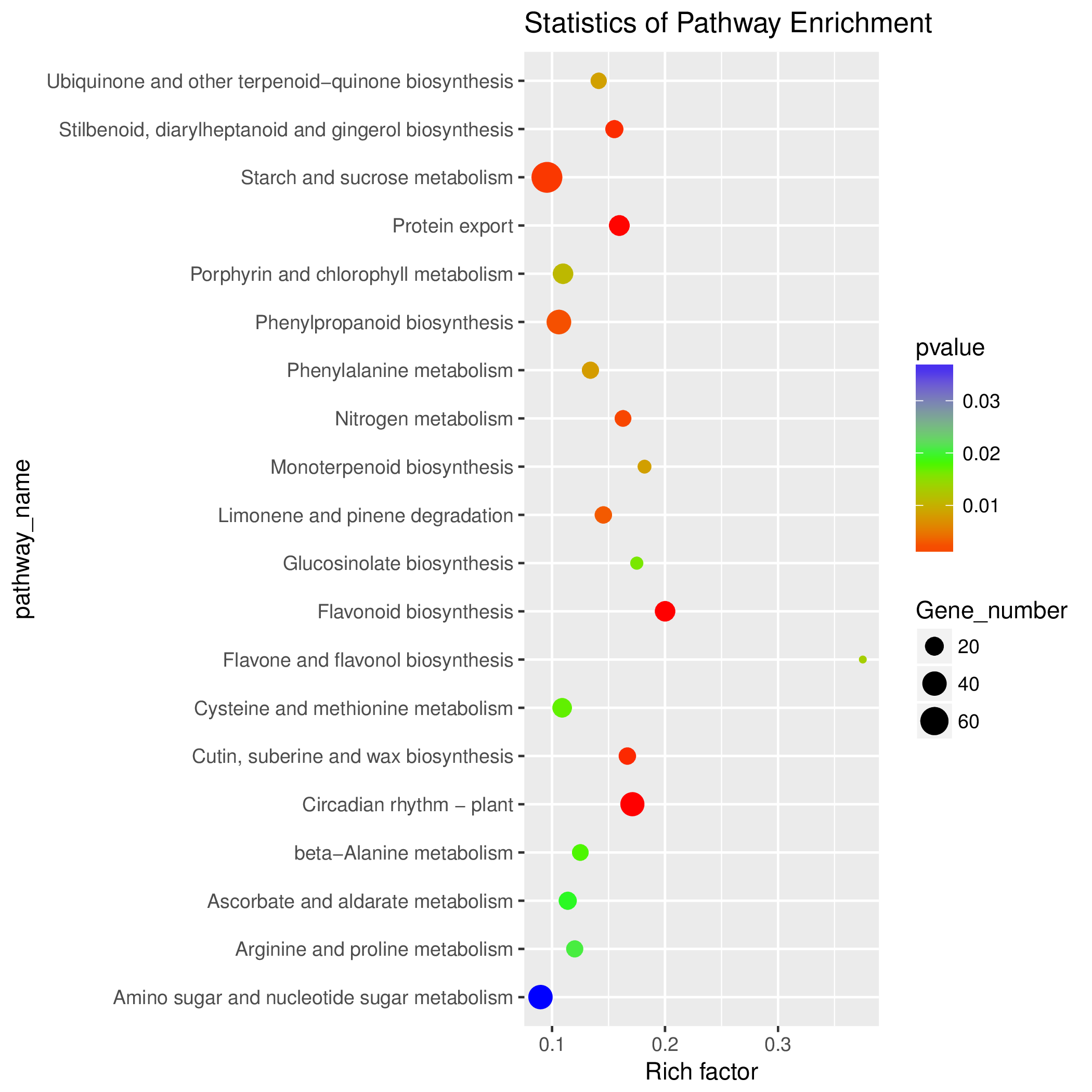


**Figure S3** Pathway enrichment analysis of DEGs obtained in the SAM of mutant *ebm3* and wild-type line ‘FT’ by transcriptome profiling.
